# Similar, but different: structurally related azelaic acid and hexanoic acid trigger differential metabolomic and transcriptomic responses in tobacco cells

**DOI:** 10.1186/s12870-017-1157-5

**Published:** 2017-11-29

**Authors:** Arnaud T. Djami-Tchatchou, Efficient N. Ncube, Paul A. Steenkamp, Ian A. Dubery

**Affiliations:** Department of Biochemistry, University of Johannesburg, Auckland Park, 2006 South Africa

**Keywords:** Augmented defense, Azelaic acid, Hexanoic acid, Hydroxycinnamic acids, Priming

## Abstract

**Background:**

Plants respond to various stress stimuli by activating an enhanced broad-spectrum defensive ability. The development of novel resistance inducers represents an attractive, alternative crop protection strategy. In this regard, hexanoic acid (Hxa, a chemical elicitor) and azelaic acid (Aza, a natural signaling compound) have been proposed as inducers of plant defense, by means of a priming mechanism. Here, we investigated both the mode of action and the complementarity of Aza and Hxa as priming agents in *Nicotiana tabacum* cells in support of enhanced defense.

**Results:**

Metabolomic analyses identified signatory biomarkers involved in the establishment of a pre-conditioned state following Aza and Hxa treatment. Both inducers affected the metabolomes in a similar manner and generated common biomarkers: caffeoylputrescine glycoside, *cis*-5-caffeoylquinic acid, feruloylglycoside, feruloyl-3-methoxytyramine glycoside and feruloyl-3-methoxytyramine conjugate. Subsequently, quantitative real time-PCR was used to investigate the expression of inducible defense response genes: *phenylalanine ammonia lyase, hydroxycinnamoyl CoA quinate transferase* and *hydroxycinnamoyl transferase* to monitor activation of the early phenylpropanoid pathway and chlorogenic acids metabolism, while *ethylene response element-binding protein, small sar1 GTPase, heat shock protein 90, RAR1, SGT1, non-expressor of PR genes 1* and *thioredoxin* were analyzed to report on signal transduction events. *Pathogenesis-related protein 1a* and *defensin* were quantified to investigate the activation of defenses regulated by salicylic acid and jasmonic acid respectively. The qPCR results revealed differential expression kinetics and, in general (except for *NPR1, Thionin* and *PR1a*), the relative gene expression ratios observed in the Hxa-treated cells were significantly greater than the expression observed in the cells treated with Aza.

**Conclusions:**

The results indicate that Aza and Hxa have a similar priming effect through activation of genes involved in the establishment of systemic acquired resistance, associated with enhanced synthesis of hydroxycinnamic acids and related conjugates.

**Electronic supplementary material:**

The online version of this article (10.1186/s12870-017-1157-5) contains supplementary material, which is available to authorized users.

## Background

As an adaptation to harmful organisms, plants have developed sophisticated immune system strategies for defense [1–3]. The plant immune system is multilayered and includes a combination of constitutive and inducible defense mechanisms to counteract colonization by microbial pathogens [1, 4]. In addition, inducible plant responses triggered by non-self recognition of common microbial structures (microbial/pathogen-associated molecular patterns, M/PAMPs) and by highly pathogen-specific effectors, which lead to M/PAMP-triggered and effector-triggered immunity (M/PTI and ETI) respectively, ensure the basis of plant resistance [3, 4]. Generally, when the basal resistance fails to prevent the entry of pathogens, plants activate another chain of defense responses called pathogen-induced resistance. Systemic acquired resistance (SAR) and induced systemic resistance (ISR) are two forms of pathogen-induced resistance wherein plant defenses are pre-conditioned by prior infection or pathogen exposure, resulting in enhanced resistance to subsequent pathogen attack [5].

Plants can be sensitized by an initial pathogen attack, treatment with pathogen-derived molecules such as M/PAMPs or even natural or synthetic compounds. This results in more rapid and intense mobilization of defense responses, thereby leading to enhanced resistance [3, 6, 7]. Plants that have been exposed to a priming process shown the ability to exhibit a defense state that can be maintained, thus having a form of ‘plant immunological memory’ [8–10]. Priming of defense thus complements innate immunity and contributes to increased resistance during SAR [7, 11–13].

As priming agents, many natural compounds including amides, aromatic compounds, carboxylic acids, glycosides, oligosaccharides and vitamins tend to be better tolerated by plants than most of the tested synthetic compounds [11, 14]. Azelaic acid (Aza) is a nine carbon dicarboxylic acid that acts as a natural inducer of plant defenses by means of a priming mechanism [15]. Aza is a derivative of oleic acid or the desaturated derivatives, linoleic and linolenic acid, but the biosynthesis pathway is largely unknown [16]. In *Arabidopsis* plants, Aza was suggested to induce SAR by priming salicylic acid (SA) biosynthesis which resulted in a faster and stronger induction response to pathogen inoculation with higher accumulation of SA [17]. It was also shown that pathogen-induced SA accumulation and *PR1* gene expression were faster and stronger in plants treated with Aza, suggesting that the latter is a priming factor [15].

Another carboxylic acid compound not derived or related to plant defensive pathways, and with proven activity as an inducer of plant defenses, is hexanoic acid (Hxa) [18]. Hxa is a potent natural priming agent with demonstrated efficiency in a wide range of host plants and pathogens. It can induce the early activation of broad-spectrum defenses by inducing callose deposition and the SA - and jasmonic acid (JA) pathways [14]. Hxa has been reported to prime tomato plants against the hemibiotrophic bacterium *Pseudomonas syringae* pv. tomato and against necrotrophic *Botrytis cinerea* [18]. Hxa treatment has also been found to protect *Arabidopsis* plants against *B. cinerea* with changes in the JA-signaling pathway upon infection [19]. The induction of many genes that characterize the Hxa priming effect, especially those related to defense, the signaling network and oxidative stress control, was revealed by microarray data of Hxa-treated plants [14]. As such, the aim of this study was to follow an untargeted metabolomics approach to investigate and compare the priming-related responses in *Nicotiana tabacum* cells triggered by Aza and Hxa.

## Results

### Metabolomic analyses

Metabolomics aims to detect and identify the chemical compounds that drive and participate in biological processes. As such, a metabolomic approach was used to compare the effect of Aza to Hxa in an attempt to qualitatively and quantitatively evaluate the biochemical effects on the cells at the metabolite level. Thus, the two priming agents were exogenously applied to the cells 3 d after sub-culturing and harvested at treatment time-points of 0, 6, 12 and 24 h. The liquid chromatography coupled to mass spectrometry (LC-MS)-based metabolomic workflow was then carried out, generating data that was analyzed by multivariate statistics and chemometric modeling.

### Chromatographic analysis

Representative chromatograms acquired using ultra-high performance liquid chromatography coupled to a quadrupole time-of-flight mass spectrometer (UHPLC-qTOF-MS) in electrospray ionization (ESI) negative mode are presented as, Additional file 1: Figure S1 and Additional file 2: Figure S2. Visual inspection of the base peak intensity (BPI) chromatograms indicated the responsiveness of the cells to the treatments over a 24 h period. Previous metabolite fingerprinting studies of phenolic compounds and -derivatives have mostly been carried out in ESI negative ionization mode [20, 21]. As such, only these data sets were processed for further analyses. Multivariate data models were generated to reveal the underlying differences in the dynamic metabolic responses of tobacco cells treated with Aza and Hxa, respectively.

### Multivariate data analysis

**Fig. 1:**
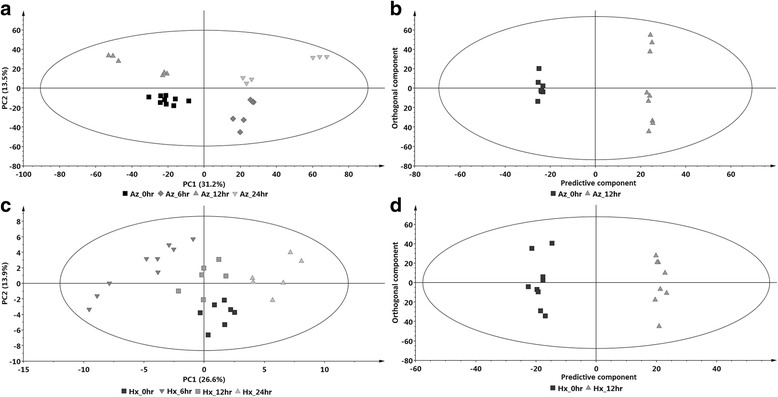
Multivariate data models depicting the metabolomic perturbations in tobacco cells in response to elicitation with 100 μM azelaic acid (Aza) and hexanoic acid (Hxa). **a** Principal components (PC1 vs. PC2) analysis shows clustering, based on differences in the metabolomes, at 0, 6, 12 and 24 h post-treatment, and (**b**) OPLS-DA score plots (0 vs. 12 h) of analytes present in extracts for the of Aza-treated cells. Equivalent graphs for extracts from Hxa-treated cells are shown in (**c**) and (**d**) respectively. The x-axes of the OPLS-DA score plots correspond to the predictive component t [1] whereas the y-axes represents the orthogonal component, to[1]. The ellipse represents Hoteling’s T2 at 95% confidence interval

The data matrices obtained from the MarkerLynx XS™ software (Waters Corporation, Manchester, UK) were exported to SIMCA (Soft Independent Modelling of Class Analogy) software, version 14 (Umetrics, Umeå, Sweden) for multivariate data analysis (MVDA). Generated models include principal component analyses (PCA)-derived score – and loadings plots, and orthogonal projection to latent structures-discriminant analysis (OPLS-DA)-derived score plots and S-plots. The former shows co-variation and correlation among the samples whereas the latter allows for the extraction of the significant biomarkers responsible for the differences between the various treatment groups (i.e. metabolites perturbed by or associated with the Aza and Hxa treatments) [22–24]. As such, the PCA score plots are presented in Fig. 1a and c, whereas the OPLS-DA score plots are presented in Fig. 1b and d and compare the 0 and 12 h time points. The models are statistically significant since the PCA plots explain total variation (R^2^X) and have predictability power (Q^2^X) > 50%, and the generated OPLS-DA plots have a cross-validated (CV) Anova *p*-value of <0.05 [22–24]. Clustering of the same treated groups at different time-points can be seen in Fig. 1a and c, indicating that the cells respond to both Aza and Hxa treatments in a time-dependent manner reflective of ongoing metabolic events.

**Fig. 2:**
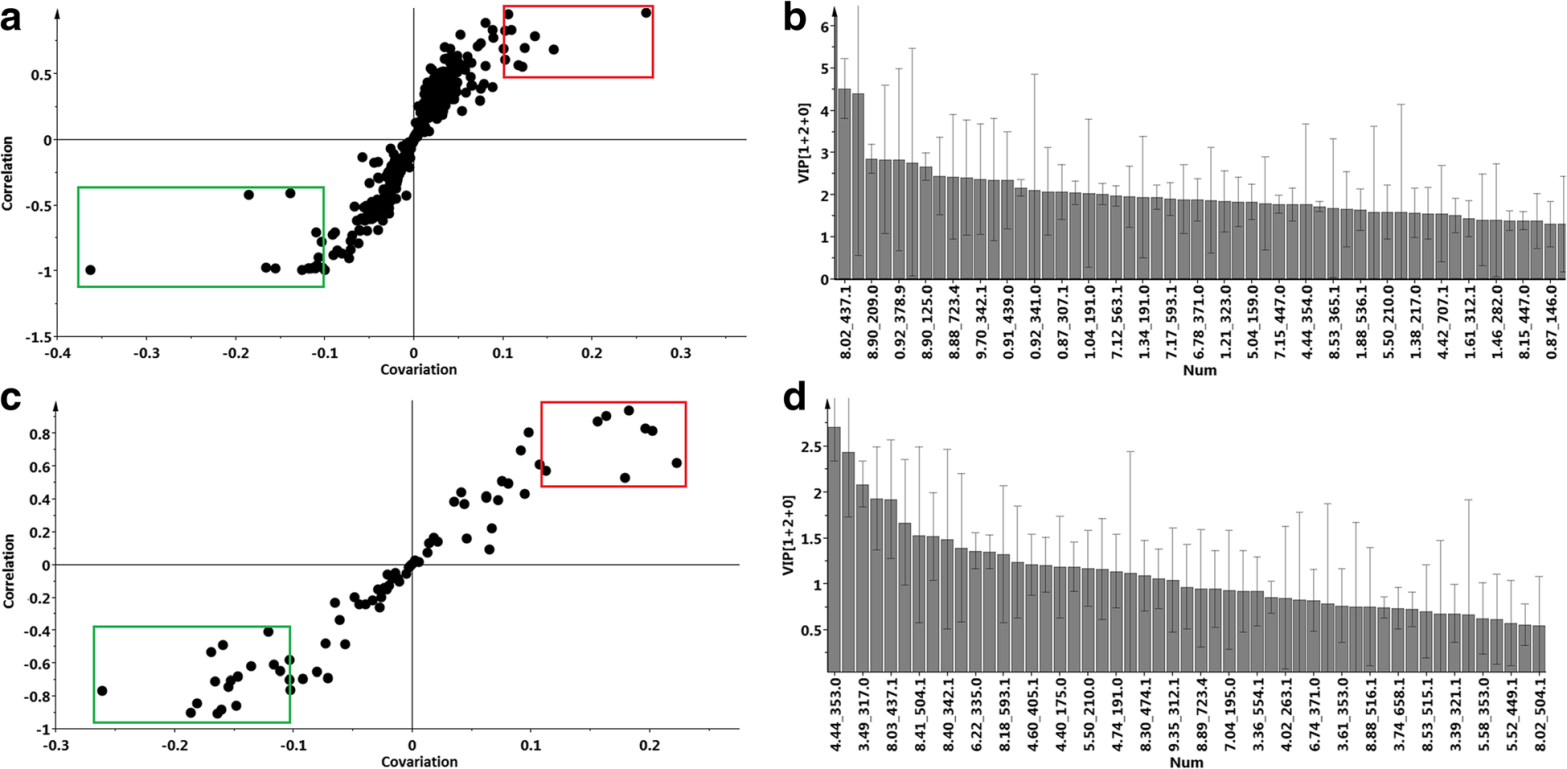
Identification of signatory biomarkers associated with the response of tobacco cells to 100 μM of the inducing agents azelaic acid (Aza) and hexanoic acid (Hxa). Shown are the (**a**) OPLS-DA S-plots and (**b**) VIP plots of analytes (Rt and *m/z* values) present in extracts derived from 12 h Aza-treated cells in comparison to the 0 h controls. Equivalent graphs for extracts from Hxa-treated cells (0 h vs. 12 h) are shown in (**c**) and (**d**) respectively. The x-axes of the OPLD-DA S-plots represent the modeled covariance or p [1] and the y-axes the modeled correlation or p(corr) [1]. The rectangles indicate the extraction of ions representing significant biomarkers (cut off: ± 0.10) responsible for the difference between the control and treated cells. The VIP plots are annotated with Rt and *m/z* values

**Fig. 3:**
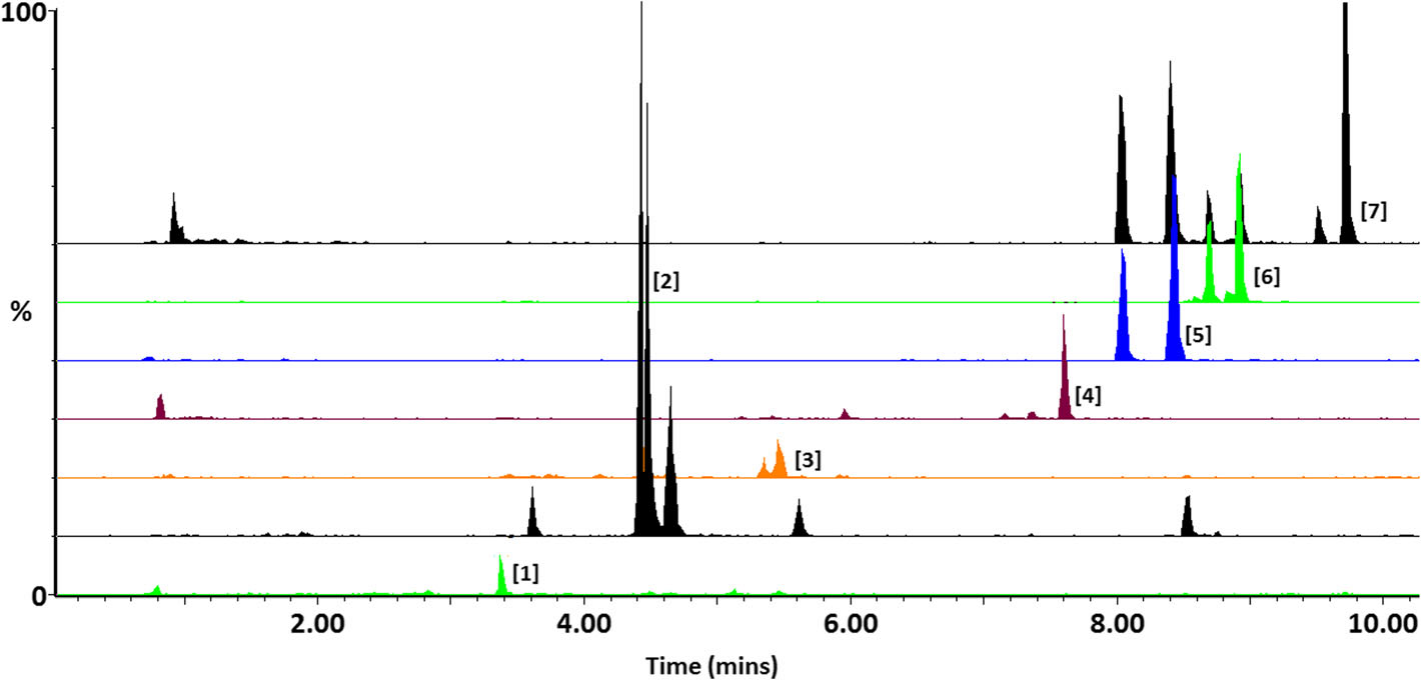
UHPLC-QTOF-MS chromatograms (extracted ion traces in ESI negative mode) indicating the chromatographic separation and mass spectrometric detection of azelaic – and hexanoic acid responsive biomarkers in extracts corresponding to the 12 h treatments: [1] caffeoylputrescine glycoside, [2] *cis*-5-caffeoylquinic acid, [3] feruloylglycoside, [4] azelaic acid glycoside, [5] feruloyl-3-methoxytyramine glycoside, [6] feruloyl-3-methoxytyramine conjugate and [7] feruloyl-3-methoxytyramine. The numbers of the structures correspond to those in Additional file 3: Figure S3 and Table 1. Perturbations in the levels of these metabolites are shown in Figs. 4 and 5

The respective S-plots of the OPLS-DA score plots were generated (Fig. 2a and c). The selected ions at the top right corner (in the red rectangle) are positively correlated to the treatment whereas the selected ions at the bottom left corner (in the green rectangle) are negatively correlated to the treatment. Extracted ions representing significant biomarkers (cut off: ± 0.10) responsible for the difference between the control and treated cells were 11 and 9 for Aza and Hex respectively. Variable importance in projection (VIP) plots (Fig. 2b and d) were also generated to confirm the significance of the selected ions, where a *p* > 1.0 on the y-axis was set as the minimum value for relevance [6, 25, 26]. Subsequently, these biomarkers were putatively annotated as listed in Table 1. A compilation of the various extracted ion chromatograms (EICs) for bio-markers **1** to **7** are presented in Fig. 3.

**Table 1:** List of tentatively annotated biomarkers associated with the pre-conditioned state in cultured tobacco cells treated with 100 μM azelaic - and hexanoic acid respectively as priming agents

Rt (min)	Ion *(m/z)*	Annotated compound	Diagnostic fragments (*m/z*)	Azelaic acid	Hexanoic acid
3.37	411.1908	[1] Caffeoylputrescine glycoside	249.1246, 321.1380, 135.0438	x	x
4.43	353.0583	[2] *cis*-5-Caffeoylquinic acid	191.0634, 135.0376	x	x
5.35	355.1017	[3] Feruloylglycoside	193.0493	x	x
7.58	349.1384	[4] Azelaic acid glycoside	187.0881	x	
8.34	504.1725	[5] Feruloyl-3-methoxytyramine glycoside	342.1246, 178.0354	x	x
8.90	546.1877	[6] Feruloyl-3-methoxytyramine conjugate	342.1443, 178.0786	x	x
9.70	342.1443	[7] Feruloyl-3-methoxytyramine	178.0532	x	

Note: the mass to charge ratio of the ions are customarily presented as m/z in italics

### Annotation of biomarkers

The tentative annotation of the signatory biomarker metabolites that were correlated with the treatment of Aza and Hxa was carried out as previously described, by comparing the fragmentation patterns of the spectra to published literature [27, 28]. Accurate annotation of structurally similar isomers of hydroxycinnamic acid (HCA) derivatives has proven to be a difficult task. However, LC-MS was chosen as the analytical platform as it is capable of discriminating between the structurally similar positional and geometric isomers of HCA derivatives [21, 29]. In addition, the LC-MS based hierarchical key developed by Clifford et al. [20] facilitated the identification of chlorogenic acids (CGAs). As a result, a total of 7 biomarkers were characterized and putatively annotated as shown in Table 1 with structures in Additional file 3: Figure S3.

### Characterization of the annotated biomarkers

The MS fragmentation patterns of the mentioned biomarkers are supplied in the Additional file 4: Figure S4 and Additional file 5: Figure S5 respectively. Biomarker **1** at Rt 3.37 min was putatively annotated as caffeoylputrescine glycoside (412 Da) with a peak at *m/z* 411.1667 [M-H]^−^ and a base peak at *m/z* 249.1153 [M-H-caffeoylputrescine-162]^−^ due to the loss of a glucosyl moiety (162 Da). The other MS-produced fragments were seen at *m/z* 321.1487 and *m/z* 135.0402 [M-H-caffeoyl-COO]^−^ that represent a decarboxylated caffeic acid moiety. Biomarker **2** at Rt 4.43 min was putatively annotated as *cis*-5-caffeoylquinic acid (354 Da) with a [M-H]^−^ peak at *m/z* 353.0735 and a base peak at *m/z* 191.0412 [M-quinic acid-18]^−^ due to the loss of a dehydrated quinic acid moiety. The other MS-produced fragment observed at *m/z* 135.0376 [M-H-caffeoyl-COO]^−^ represents a decarboxylated caffeic acid moiety. The putative annotation of these molecules, considering both regio- and geometric isomerization, was based on the hierarchical diagnostic fragmentation as previously described [20]. Accordingly, the molecule was annotated as *cis*-5-caffeoylquinic acid because of elution order and the presence of the base peak at *m/z* 191, indicative of a 5-acyl position [30]. Biomarker **3** at Rt 5.35 min was annotated as feruloylglycoside (356 Da) with a [M-H]^−^ peak at *m/z* 355.0933 and a base peak at *m/z* 193.0430 [M-H-ferulic acid]^−^ due to the loss of a glucosyl moiety (162 Da) [21]. Biomarker **4** was annotated as azelaic acid glycoside (350 Da) with a [M-H]^−^ peak at *m/z* 349.1389 and a base peak at *m/z* 187.0883, indicative of azelaic acid [M-H-azelaic acid-162]^−^ due to the loss of a glucosyl moiety (162 Da). Expectedly, this ion is only present in the azelaic acid-treated samples (Table 1). Biomarker **5** at Rt 8.34 min was annotated as feruloyl-3-methoxytyramine glycoside (505 Da) with a peak at *m/z* 504.1638 [M-H]^−^ and a base peak at *m/z* 342.1240 [M-H-feruloyl-3-methyltyramine-162]^−^ due to the loss of a glucosyl moiety (162 Da). Biomarker **6** at 8.90 min was annotated as feruloyl-3-methoxytyramine conjugate (547 Da) with a peak at *m/z* 546.1868 [M-H]^−^ and a base peak at *m/z* 342.1231 [M-H-feruloyl-3-methoxytyramine-203]^−^ due to the loss of a conjugate of 203 Da. Biomarke**r 7** at 9.70 min was annotated as feruloyl-3-methoxytyramine (343 Da) with a base peak at *m/z* 342.1231 [M-H-feruloyl-3-methyltyramine]^−^. The annotation of the feruloyl-methoxytyramine conjugated molecules (biomarkers **5, 6** and **7**) were as previously described [27, 28].

### Relative quantification of annotated biomarkers

**Fig. 4:**
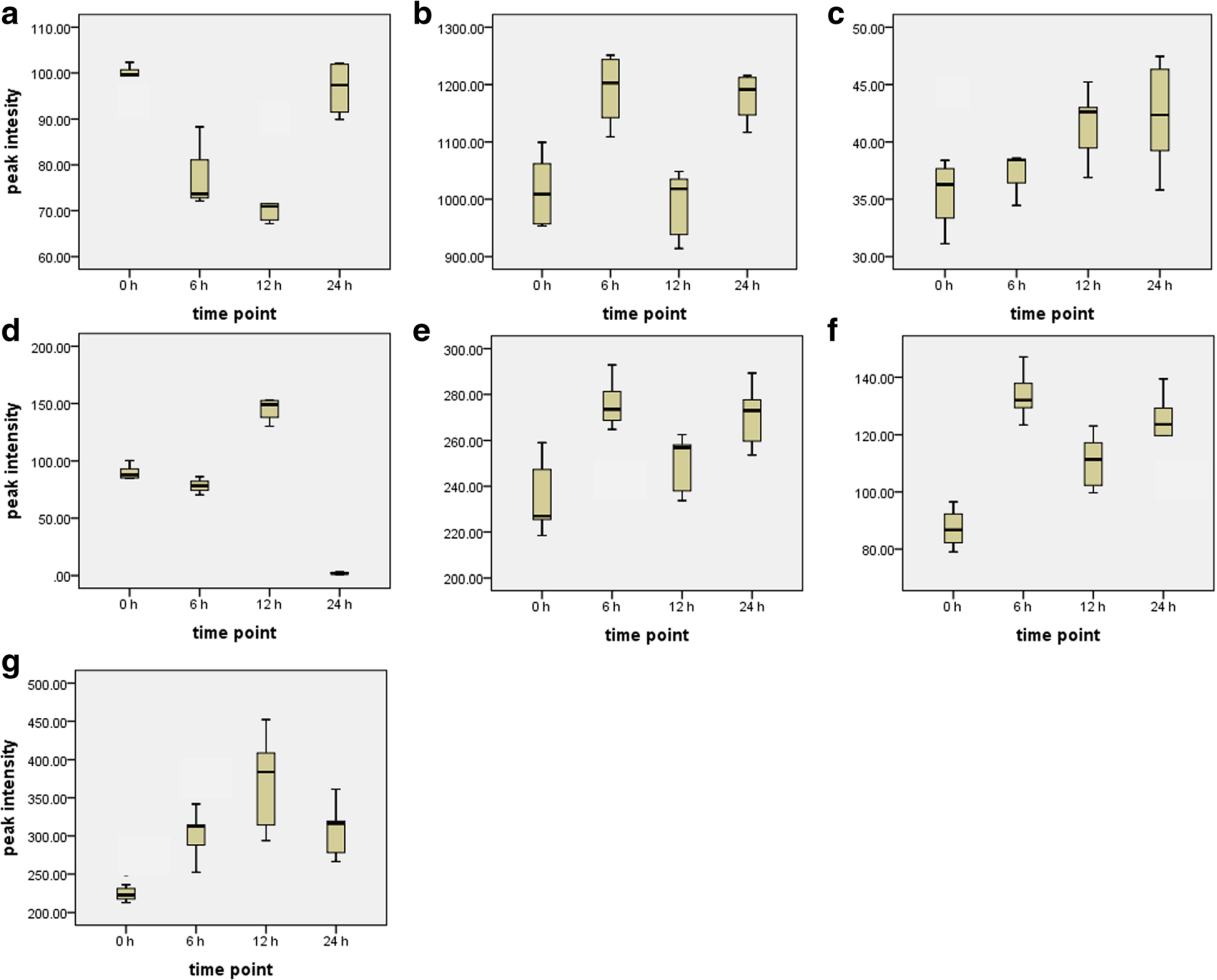
Box-and-whiskers plots of time-dependent changes in the levels of signatory biomarkers identified in extracts from tobacco cells treated with 100 μM azelaic acid. Shown are the relative concentrations of (**a**) caffeoylputrescine glycoside [1], (**b**) *cis*-5-caffeoylquinic acid [2], (**c**) feruloylglycoside [3], (**d**) azelaic acid glycoside [4], (**e**) feruloyl-3-methoxytyramine glycoside [5], (**f**) feruloyl-3-methoxytyramine conjugate [6], and (**g**) feruloyl-3-methoxytyramine [7]. The numbers of the structures correspond to those in Additional file 3: Figure S3 and Table 1. Values represent the mean of three biological replicates and three technical replicates, *n* = 9)

**Fig. 5:**
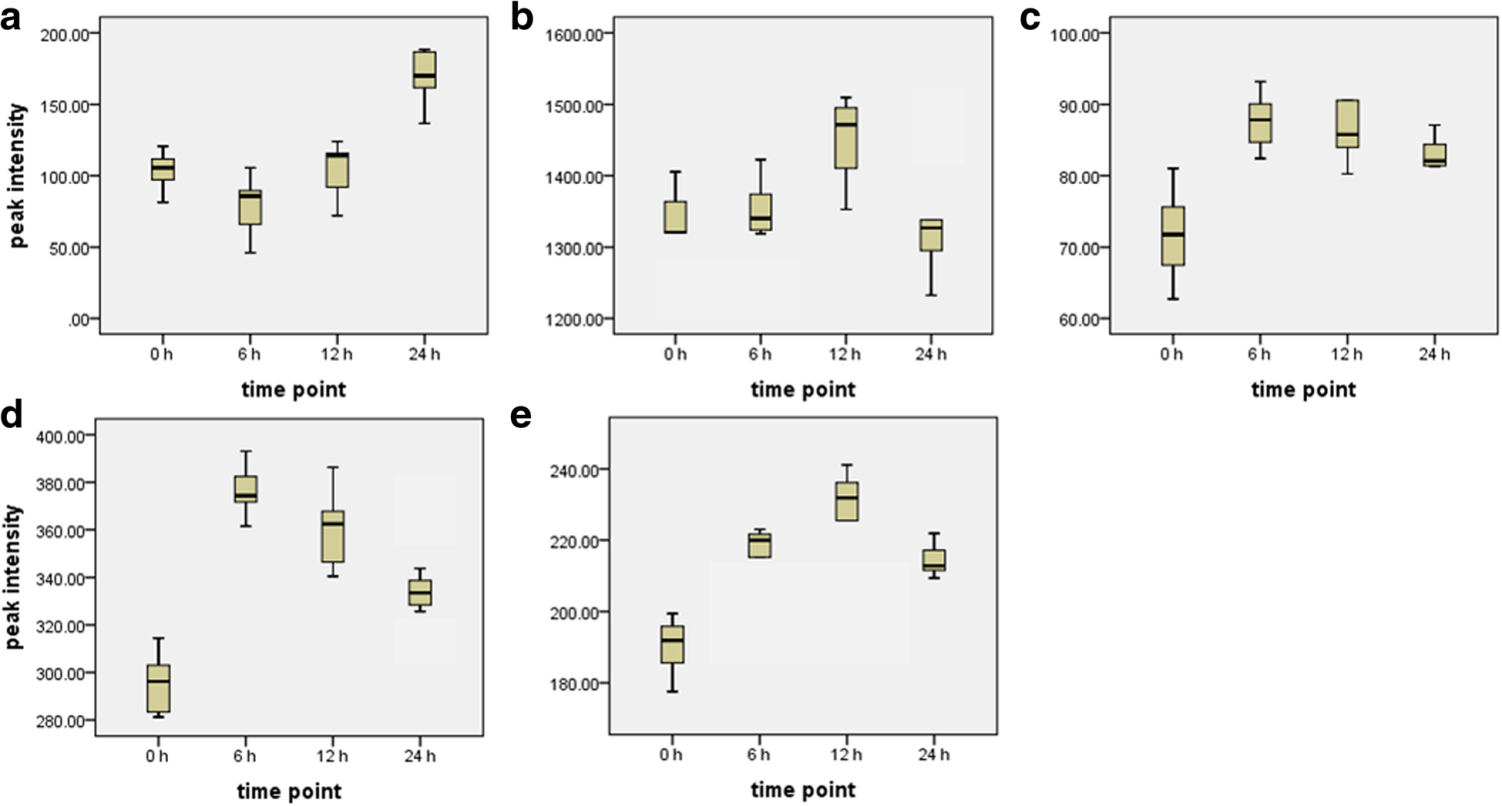
Box-and-whiskers plots of time-dependent changes in the levels of signatory biomarkers identified in extracts from tobacco cells treated with 100 μM hexanoic acid. Shown are the relative concentrations of (**a**) caffeoylputrescine glycoside [1], (**b**) *cis*-5-caffeoylquinic acid [2], (**c**) feruloylglycoside [3], (**d**) feruloyl-3-methoxytyramine glycoside [5]  and (**e**) feruloyl-3-methoxytyramine conjugate [6]. The numbers of the structures correspond to those in Additional file 3: Figure S3 and Table 1. Values represent the mean of three biological replicates and three technical replicates, n = 9)

The box-and-whiskers plots show the relative concentrations of the annotated metabolites after different time points (0, 6, 12, and 24 h post-treatment) in the Aza- and Hxa-treated samples (Figs. 4 and 5). This relative quantitative analysis (based on peak intensities) shows a general trend of an increase in concentration from 0 to 24 h post-treatment, with some metabolites having the highest relative concentrations at 6, 12 or 24 h. The differential effect on metabolite kinetics is illustrated in the case of compound [1], caffeoylputrescine, where the relative levels were found to decrease initially in Aza-treated cells between 0 and 12 h, but recovered to basal levels by 24 h. In the case of the Hxa-treated cells, there was a slight decrease at 6 h, followed by an increase greater than basal levels up to 24 h.

### Gene expression analysis

**Fig. 6:**
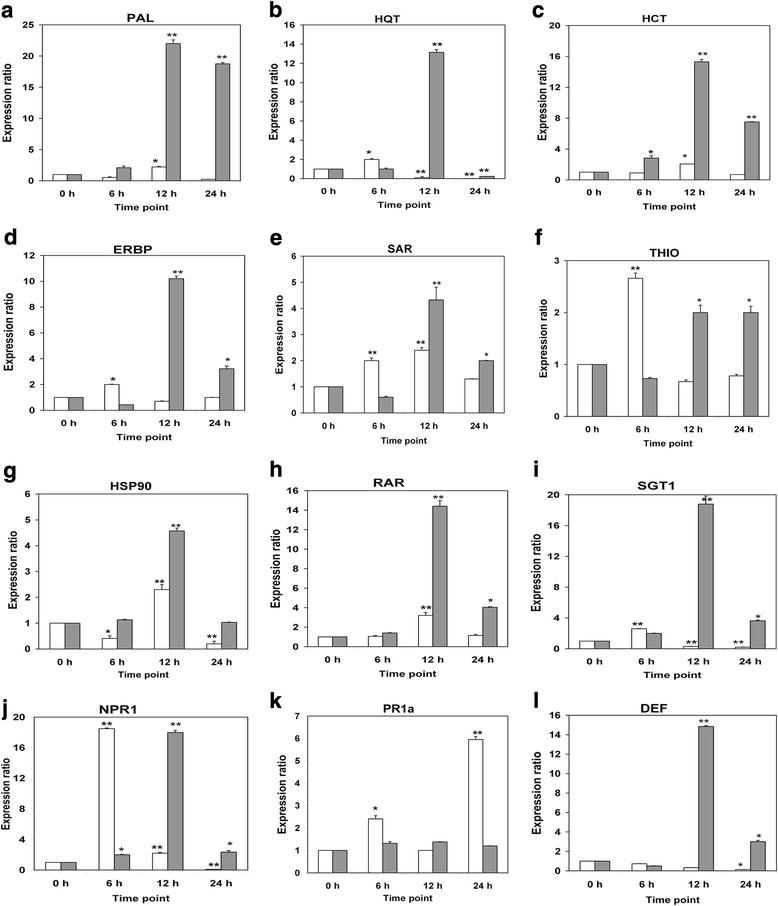
Differential gene expression analysis in *Nicotiana tabacum* cells following induction with Aza (in white) and Hxa (in grey). The data was normalized using *Elf α* and *18S* to give the relative gene expression where the error bars represent the standard error of mean. **a** *Phenylalanine ammonia lyase (PAL),* (**b**) *Hydroxycinnamoyl-CoA:quinate hydroxycinnamoyl transferase (HQT),* (**c**) *Hydroxycinnamoyl-CoA:shikimate/quinate hydroxycinnamoyl transferase (HCT),* (**d**) *Ethylene response element-binding* protein (EREBP), (**e**) *Small Sar1 GTPase* (*SAR1-GTPase),* (**f**) Thioredoxin (THIO), (**g**) *Heat shock protein 90 (HSP90),* (**h**) *Required for Mla12 Resistance (RAR1)*, (**i**) *Suppressor of the G2 allele of* Skp1 (SGT1), (**j**) *Non-expressor of PR genes 1 (NPR1),* (**k**) *Pathogenesis-related protein 1a (PR-1a)* and (**l**) *Defensin*. Increases indicated by a single asterix (*) indicates a significant difference with *p* < 0.05 and (**) indicates a highly significant difference with *p* < 0.01

In order to profile the gene expression in tobacco cells in response to the priming effect of Aza and Hxa treatments, total RNA was isolated after 0, 6, 12 and 24 h elicitation. Quantitative expression analysis of twelve genes which included: (a) *Phenylalanine ammonia lyase (PAL),* (b) *Hydroxycinnamoyl-CoA:quinate hydroxycinnamoyl transferase (HQT),* (c) *Hydroxycinnamoyl-CoA:shikimate/quinate hydroxycinnamoyl transferase (HCT),* (d) *Ethylene response element-binding protein (EREBP),* (e) *Small Sar1 GTPase (SAR1-GTPase),* (f) *Thioredoxin (THIO),* (g) *Heat shock protein 90 (HSP90),* (h) *Required for Mla12* Resistance (RAR1), (i) Suppressor of the G2 allele of Skp1 (SGT1), (j) *Non-expressor of PR genes 1 (NPR1),* (k) *Pathogenesis-related protein 1a (PR-1a)* and (l) *Defensin*, was performed as previously described [7] and normalized against *Elf α* and *18S rRNA* to give the relative gene expression. The results (Fig. 6 a-l) indicate that the transcripts exhibited different expression kinetics that can be described as early- (6 h), mid- (12 h) and late- (>12 h) responses. The fold-expression varied from relatively low (>2 fold) to high (>10 fold), compared to the basal levels of non-treated cells. In general, except for *NPR1*, *Thionin* and *PR1a*, the relative gene expression ratios observed in the Hxa treated cells was significantly greater than the expression observed in the cells treated with Aza.

## Discussion

Priming shares some metabolic features with acquired/induced resistance with the aim of faster and stronger activation of stress-inducible defense reactions upon a subsequent pathogen challenge [31]. Since investigations of priming in plants started with molecular tools, it has improved the understanding of the activation of the plant innate immune system through the identification of defense genes and analysis of signaling pathways leading to SAR and ISR. Many natural and synthetic compounds have been shown to be critical regulators of acquired/induced resistance by means of a priming mechanism, or as direct activators of induced immunity [11, 13, 14]. In addition to the acceleration of the plant’s ability to activate defense responses, the emerging picture is that priming represents an important adaptation or survival mechanism in plants to cope with abiotic and biotic stresses [10, 13, 32]. Although recent results started to unravel the molecular mechanism behind priming, it is still relatively poorly understood. Within this context the present study was undertaken to perform a comparative metabolomics investigation regarding the mode(s) of action and effectiveness of Aza and Hxa as inducers of resistance in *N. tabacum* cells in support of priming, with the focus on genes and secondary metabolites likely to be involved in host defense. Our findings revealed that the response of cells to treatment with Aza and Hxa is time-dependent, with differential effects on the up- or down-regulation of metabolites as well as altered expression kinetics of selected genes involved in the activation and execution of the plant defense response.

### Metabolomic analyses

Metabolomics is a qualitative and/ or quantitative approach for the analysis of metabolites under certain physiological states in a biological system [33, 34]. In essence, as the ultimate recipients of biological information flow in cells, the spectrum and level of metabolites not only play a crucial role in the expression of genes and stability of proteins, but also determines the phenotypic properties of the cell or organism [35]. Ultimately, this approach significantly contributes to the understanding of unassigned and unidentified compounds from undefined metabolic pathways [36, 37]. Metabolomics has therefore been used in various recent studies that reported on the physiological processes of plant stress biology, the plant immune system and plant-microbe interactions. The exogenous application of priming agents to plant tissues or cultured cells has shown to be effective models to investigate up- or down-regulation of genes involved in defense-related cellular pathways [38–40]. In this way plant bioactive secondary metabolites resulting from these pathways can be rapidly biosynthesized, extracted and analyzed [27, 28, 41].

### Secondary metabolites and the defensive roles of the annotated metabolites

Secondary plant metabolites constitute a formidable contributor to the chemical defenses of plants, either as phytoanticipins or phytoalexins [40]. These metabolites include phenylpropanoids, terpenoids, alkaloids and glucosinolates, depending on the particular species [34, 36, 42]. These metabolites play an important role in plant defense systems and environmental adaptation, and their presence fluctuates in response to different environmental stimuli [9, 27, 28]. The knowledge on the accumulation of secondary metabolites in response to infection, has been utilized to study the underlying biochemical responses to stress [9, 27, 40].

The obtained results indicate that the exogenous treatment of tobacco cells with both Aza and Hxa led to the production of mainly HCA compounds (conjugates of caffeic acid and ferulic acid) listed in Table 1. This is in accordance with our previous results [27, 28]. PAL is the key enzyme at the entry point of the phenylpropanoid pathway, leading to the production of cinnamic acid that is further metabolized to various phenolic compounds [43]. Noteworthy, in Fig. 6, there is up-regulation of *PAL* gene transcripts post 12 h Aza and Hxa elicitation. As a major part of the phytoalexins, phenylpropanoid compounds play a huge role in plant defense against stressors such as hostile environmental conditions [44–46]. In addition, biological functions of these compounds include structural support (phenylpropanoid-based polymers) and signaling in plant defense [33, 44]. As such, priming of plants by biotechnological induction of the phenylpropanoid pathway is an effective means of increasing resistance against stress conditions.

HCA-related metabolites have been shown to be widely distributed in plants and compounds such as caffeic acid and ferulic acid naturally occur either in conjugated or non-conjugated forms. Solanum species such as *N. tabacum, S. tuberosum* and *S. lycopersicum* are known to produce HCA ester and - amide conjugates. Putrescine, a polyamine, often conjugates to HCAs to give rise to compounds such as caffeoylputrescine and feruloylputrescine [47, 48]. The decarboxylation of tyrosine results in tyramine which is also often conjugated to HCAs upon pathogen infection [49]. In addition, an ester bond can be formed between HCAs and a quinic acid to result in compounds known as chlorogenic acids [9, 27, 28, 45].

In this context, caffeoylputrescine glycoside (biomarker **1**) was found to be induced by Hxa whereas the levels of *cis*-5-caffeoylquinic acid (biomarker **2**), feruloylglycoside (biomarker **3**), feruloyl-3-methoxytyramine glycoside (biomarker **5**), feruloyl-3-methoxytyramine conjugate (biomarker **6**) and feruloyl-3-methoxytyramine (biomarker **7**) increased after either 6 or 12 h of post-elicitation with both Aza and Hxa (Fig. 4). In comparison, there was an increase of azelaic glycoside (biomarker **4**) at 6 h and further at 12 h, and a decrease at 24 h following Aza elicitation. This is indicative that upon uptake and accumulation of Aza, the cells conjugated the molecules with glucose, possibly as a detoxification mechanism or for storage purposes.

Although quantitatively different, the effect of Hxa as a priming agent is qualitatively similar to that of Aza with regard to up-regulation of the early phenylpropanoid pathway, resulting in the biosynthesis of similar derivatives of caffeic acid and ferulic acid. The dynamic responses can be attributed to active metabolism, including synthesis, interconversion and degradation of stored conjugates as a mechanism to rapidly supply demands for HCAs [9].

### Quantitative gene expression analysis

Quantitative expression analyses of twelve genes which included: (i) *PAL, HQT* and *HCT*; (ii) *EREBP, SAR1-GTPase* and *THIO;* (iii) *HSP90, RAR1* and *SGT1*; (iv) *NPR1, PR-1a* and *Defensin*, were performed. The results indicate that the transcripts, associated with the various functional categories discussed below, exhibited different expression kinetics that can be described as early, mid and late responses.

### Metabolism

Activation of signal transduction networks after pathogen recognition results in reprogramming of cellular metabolism, which leads to a large change in gene activity. Phenylalanine is synthesized via the shikimate pathway that also leads to the synthesis of quinic acid. PAL is the enzyme involved in the deamination of phenylalanine to *trans*-cinnamic acid, thus linking primary metabolism to secondary metabolism [43, 50]. The quinic acid pool acts as a reservoir that can be reversibly injected into the main pathway for esterification reactions with HCAs [9, 27]. The production of *trans*-cinnamic acid (and the HCA derivatives) occurs in response to stress-induced increases in PAL activity, and this represents the first step in the biosynthesis of various phenylpropanoids involved in plant defense: hydroxylated and methoxylated cinnamates, chlorogenic acids, coumarins, flavonoids and lignin precursors.

We investigated the expression of *PAL, HCT* and *HQT.* The expression of *PAL* was significantly up-regulated at 12 h in the cells treated with Aza as well as at 12 and 24 h in the cells treated with Hxa (Fig. 6a). HQT is one of the crucial enzymes for the synthesis of 5-caffeoylquinic acid (a CGA), catalyzing the transesterification reaction of caffeoyl-CoA with quinic acid [51]. CGAs and related derivatives exhibit radical scavenging activity and have been identified as phytoanticipins and resistance biomarkers, as well as non-antimicrobial defense compounds that interferes with infection processes [9, 27, 28, 52]. The expression of *HQT* was significantly up-regulated at 6 h in the cells treated with Aza and at 12 h in those treated with Hxa (Fig. 6b). HCT functions in an alternative route to CGA synthesis where *p*-coumaroyl-CoA is first *trans*-esterified with quinic acid before hydroxylation to yield 5-caffeoylquinic acid and, similarly, plays a critical role in the phenylpropanoid biosynthetic pathway [53]. Our study showed that following Hxa treatment of the cells, the expression of *HCT* was significantly up-regulated from 6 to 24 h, with maximum expression at 12 h (Fig. 6c). In cells treated with Aza, *HCT* was significantly up-regulated only at 12 h. Based on the differential expression of the *PAL, HQT* and *HCT* genes, we suggest that the priming action of Aza and Hxa involves activation of the early shikimate/phenylpropanoid pathway in support of the chemical defenses associated with disease resistance in plants.

### Signal perception and transduction

Priming for enhanced defense against biotic/abiotic stress requires specific cellular signaling components upon treatment with an inducing agent. *SAR1-GTPase* (a small monomeric GTP-binding protein belonging to the *Rho* subfamily), associated with plant signaling events, was significantly up-regulated in the tobacco cells treated with Aza from 6 to 12 h as well as cells treated with Hxa from 12 to 24 h. The maximum expression was observed in the cells treated with both Aza and Hxa at 12 h (Fig. 6e). SAR1-GTPase acts as a molecular switch and is involved in intracellular signaling pathways downstream of inducible lectin domain receptor-like kinases, possible receptors for recognition of extracellular pathogen-derived P/MAMPs [54, 55]. As such, treatment with Aza and Hxa triggered the expression of *SAR1-GTPase* which plays a positive role in plant immunity as seen previously in the tobacco cells treated with isonitrosoacetophenone (INAP) [7]. The results indicate that Aza triggered the expression of *SAR1-GTPase* earlier than Hxa, but with the latter lasting longer than the Aza effect. However as stated, with both treatments the highest expression level of *SAR1-GTPase* was observed at 12 h. The results indicate that the mode of action of Aza might be similar to that of Hxa on this key gene.

### Transcription factors

Transcription factors play an important regulatory function in the onset of priming [56]. EREBP is known to be involved in transcriptional activation and in the cells treated with Aza an up-regulation was observed at 6 h post-elicitation. Up-regulation was also observed for *EREBP* transcripts in the cells treated with Hxa at 12 and 24 h, with a maximum 10-fold expression at 12 h (Fig. 6d). The results thus indicated that Aza triggered the expression of *EREBP* earlier than Hxa, but with the latter effect lasting longer and with the highest expression level observed at 12 h. Previous findings revealed that, during plant-pathogen interactions, the rate of ethylene biosynthesis (which is mediated by EREBP) increases rapidly and is linked to the induced transcription of some basic-type *PR* defense genes [57]. Therefore, EREBP plays an important regulatory role in the onset of priming as also observed previously in *N. tabacum* cells primed with INAP [7]. Since some defense mechanisms are not directly activated upon induction of the primed state, it can be assumed that the up-regulated expression of *EREBP* transcripts resulted in the activation of the defense response that is marked by the transcription of the appropriate defense-related genes.

### Molecular chaperones

The HSP90, SGT1 and RAR1 proteins form a molecular chaperone complex involved in diverse biological signaling including innate immunity and *R* gene-mediated disease resistance [58]. This complex coordinately contributes to the stability of the nucleotide-binding leucine-rich repeat (NB-LRR)-containing proteins which are a group of receptors mediating innate immune responses to microbial pathogens. In addition, the HSP90-SGT1-RAR1 complex is crucial in the activation of R proteins and therefore a critical component of the plant immune response [59]. In this study, the expression pattern of *HSP90, RAR1* and *SGT1* in the cells treated with Hxa showed an up-regulation from 12 to 24 h, with a maximum expression of each gene at 12 h resulting in 4-, 18- and 14-fold increases respectively for each gene (Fig. 6 g,h,i). In the cells treated with Aza, up-regulation was observed in the expression of *SGT1* at 6 h, and *HSP90* and *RAR1* at 12 h. Similar expression patterns showing up-regulation was observed for *HSP90* and *RAR1* in the cells treated with both priming agents at 12 h. These results indicate the involvement of the HSP90-SGT-RAR1 complex in the responses triggered by Aza and Hxa treatment. Furthermore, the findings also showed that the mode of action of Aza and Hxa is similar on *HSP90* and *RAR1* at 12 h (Fig. 6 g,h), but that at other time points the effect on these gene transcripts differed.

### Response regulators

The treatment of tobacco cells with Aza and Hxa resulted in the induction of the *NPR1* gene. NPR1 is an important defense regulator protein that is required for SAR establishment [60], which is regulated by the endogenous accumulation of the signal molecule SA with NPR1 positioned at the cross-roads of multiple defense pathways [61]. Upon SAR induction a biphasic change in cellular reduction potential occurs, leading in reduction of NPR1 to a monomeric form which accumulates in the nucleus and induces the expression of some *PR* defense genes via SA pathways [60, 62, 63]. A functional *NPR1* gene is required for priming and the NPR1 protein is suggested to be one of the receptors for SA [15]. Previously we reported that *NPR1* was significantly induced in *N. tabacum* cells from 2 to 12 h, with a 10-fold increase at 8 h, following the induction of INAP as priming agent [7]. In this study, the treatment of tobacco cells with Aza led to a significant up-regulation of *NPR1* transcripts from 6 to 12 h with a maximum 18-fold increase in expression at 6 h. In comparison, the treatment with Hxa led to a notable up-regulation of *NPR1* from 6 to 24 h with a maximum expression of 18-fold increase at 12 h (Fig. 6j). Knowing that the transcripts levels of *NPR1* are responsive following SA treatment/accumulation to activate *PR*-gene expression and SAR, this suggests that the SA signaling pathway is also involved in the tobacco cellular response to Aza and Hxa treatment. Finally, the results showed a similar mode of action of Aza and Hxa to trigger enhanced expression of *NPR1*, although the maximum expression was observed at different time points.

Thioredoxins are known to function in redox signaling, thereby affecting NPR1 and thus also involved in defense responses. In addition, these enzymes act as chaperones and disulphide isomerases in protein folding of newly synthesized proteins [63]. Here, up-regulation was observed for *Thioredoxin* at 6 h in cells treated with Aza, and at 12 and 24 h in the cells treated with Hxa, indicating differential responsiveness to the two priming agents (Fig. 6f).

### Defense-related proteins

Two important gene transcripts found to be induced by Aza and Hxa are *PR-1a* and *Defensin,* the accumulation of which results upon treatment with resistance-inducing agents [15]. PR-1 proteins have routinely been used as molecular markers of SA-dependent SAR, and contribute to increase pathogen resistance by directly exerting harmful effects to microbial invaders during SAR [11]. In contrast, Defensin, that exhibits antifungal and antibacterial activity [64], is controlled by JA/ET-dependent pathways and is regarded as a marker for JA signaling [62, 65]. We have previously reported that *PR-1a* and *PR-1b* transcripts were induced during SAR establishment in tobacco cells following the priming action of INAP which confers resistance to *P. syringae* pv. *tabaci* [7]. Here, our findings also revealed that there was a significant up-regulation in the expression of *PR-1a* at 6 h and a highly significant up-regulation at 24 h in the cells treated with Aza. In contrast, the cells treated with Hxa showed only a slight / non-significant increase of the expression of *PR-1a* at all time-points (Fig. 6k). In addition, the qPCR data showed that the *Defensin* transcripts were significantly up-regulated at 6 and 12 h, with a maximum 14-fold increase in expression in cells treated with Hxa (Fig. 6l). In contrast to the Hxa action, the cells treated with Aza displayed no differential expression of *Defensin* from 6 to 12 h, but rather a significant down-regulation at 24 h. The differential changes in the transcript levels of *PR-1a* and *Defensin* indicate that the corresponding genes were responsively dissimilar to the treatments, with *PR1a* responding to Aza and *Defensin* responding to Hxa, thus implying differential actions of SA and JA/ET in the action mechanisms of the two inducers.

## Conclusions

The concept of priming can encompass various induced-resistance phenomena in plants [13] that offers protection against a wide spectrum of abiotic and biotic stresses. However, the molecular mechanisms and metabolic basis of this stress-imprinting on plant immunity is not fully elucidated, and may vary between plants from different families. In the case of *N. tabacum* and other members of the *Solanaceae,* it could depend on the controlled, dynamic balance between biosynthesis and degradation of phenolic compounds [27, 28]. The phenylpropanoid pathway with HCA intermediates represents an important link to resistance for the production of different phenolics in free or conjugated forms. Our results add further support for this viewpoint, involving the modulation and mobilization of phenolic compounds and enhanced synthesis of some of the conjugates, since both Aza and Hxa resulted in the biosynthesis of caffeoyl- and feruloyl-HCAs, conjugated to quinic acid, putrescine, tyramine and glucose. The commonalities between the biomarkers identified in response to Aza and Hxa elicitation indicates very similar changes to the respective metabolomes. This indicates a high interconnectivity between the signatory biomarkers, again demonstrating the centrality of the early shikimate/phenylpropanoid pathway in the altered metabolism due to Aza and Hxa.

This study also revealed that Aza and Hxa are able to induce several genes of importance to signaling, priming and defense-related responses in plants. At the transcriptome level, and at equimolar concentrations, Hxa generated higher transcript levels of the genes investigated. The different changes in the transcript levels of SA- and JA/ET-responsive marker genes suggest differential action mechanisms for the two inducers. However, the combined results indicate that Aza and Hxa both lead to a priming effect associated with enhanced synthesis of HCAs and related conjugates, and activation of genes involved in the establishment of acquired resistance.

## Methods

### Plant material and growth conditions

*Nicotiana tabacum* cv Samsun cell cultures [7, 9] were established from callus derived from sterile, in vitro grown tobacco plants grown from seeds (Agriculture Research Council, Roodeplaat, South Africa). For callus induction, stem and leaf sections were placed on Murashige and Skoog (MS) medium containing 0.25 mg/L 2,4-dichlorophenoxyacetic acid and 0.25 mg/L kinetin (pH 5.8). Cell suspensions were grown in liquid MS medium with the same hormone composition, whilst continuously shaking at 120 rpm in the dark at 25 °C [27, 28, 30]. Cells were sub-cultured into fresh medium every 7 d. All of the experiments were conducted 2–3 d after sub-culturing, during the logarithmic stage [66].

### Experimental design, elicitation and harvesting

Each experiment was conducted a minimum of three times in order to generate the required minimum number of biological replicates required for metabolomics analyses [35–37]. Cell suspensions, as described above, were treated with equimolar concentrations of Aza and Hxa (final concentrations of 100 μM) (Sigma Aldrich, Germany). The two inducers were prepared as 10 mM stock solutions, dissolved in MS medium and with the pH adjusted to 5.8 [7]. Naïve (non-treated / non-stimulated cells) exhibited no changes over the 24 h time period and served as a negative controls. Cells were harvested at the selected time points (0, 6, 12 and 24 h) by filtration onto 55 mm circle filter papers using a vacuum filtration system (Millipore, Billerica, MA, USA). The cells were transferred into Falcon tubes and cold, sterile MS medium (without any vitamins, hormones and inducing agents) was added to 50 mL to wash the cells free of any original culture medium. Thereafter, the cells were filtered again as described, weighed and processed for extraction.

### Metabolite extraction

The washed and filtered cells were weighed to obtain 1 g of each sample and resuspended in a volume of 10 mL (1:10 m/v) 100% methanol. The suspensions were homogenized using a probe sonicator (Bandelin Sonopuls, Berlin, Germany) set at 55% power for 15 s with 4 cycles. The homogenates were centrifuged at 5100 x *g* for 15 min at room temperature and the supernatants concentrated to approximately 1 mL on a rotary evaporator set at 50°C. The resulting volumes were transferred into 2 mL Eppendorf tubes and dried to completeness in a heating block set at 50°C overnight. The dried residues were reconstituted in 500 μL 50% (*v*/v) UHPLC-grade methanol (Romil Chemistry, Cambridge, UK) in autoclaved milli-Q water, and filtered through a 0.22 μm nylon syringe filter into 300 μL inserts fitted into glass vials with slitted caps. The filtrates were stored at −20°C until analysis. To ensure reproducibility and account for analytical variability, the obtained results were from three independent biological repeats, each analyzed in triplicate (i.e. three technical repeats) for all samples. During data acquisition, pooled samples were used for quality control (QC) checks. Sample acquisition was randomized and the QC sample analyzed every 10 injections to monitor and correct changes in the instrument response.

### Chromatographic – And mass spectrometric analyses

UHPLC-MS analyses were performed on a Waters Acquity UHPLC system coupled in tandem to a SYNAPT G1 HDMS qTOF mass spectrometer (Waters Corporation, Milford, MA, USA). The liquid chromatography was carried out on a HSS T3 C18 Acquity UHPLC column (Waters, Milford, MA, USA) thermostatted at 60 °C using a gradient elution profile over a total run time of 20 min. The binary solvents utilized consisted of UHPLC grade water (eluent A) and acetonitrile (eluent B) (Romil Pure Chemistry, Cambridge, UK), both containing 0.1% formic acid. The initial conditions were 5% A and gradient elution was introduced to change the chromatographic conditions to 90% B at 16 min and the conditions were kept constant for 1 min; the gradient was then dropped to the initial conditions and held for 2 min. The flow rate was 0.4 mL/min whilst an injection volume of 3 μL was used. Separated analytes were detected using a photodiode array detector; scanning range from 200 to 500 nm with 1.2 nm resolution and a sampling rate of 20 points/s.

The mass spectrometry component used for the detection of separated analytes was a SYNAPT G1 high definition qTOF-MS, used in V-optics and operated in the electrospray ionization (ESI) positive and negative modes. A reference calibrant of leucine enkephalin (50 pg/mL) was used to obtain typical mass accuracies between 1 and 3 mDa. The following parameters were also set up: a capillary voltage of 2.5 kV and the sampling cone voltage at 30 V, trap collision 3 V and the extraction cone at 4 V. The scan time was 0.1 s covering the 100 to 1000 Da range. The source temperature was 120 °C whereas the desolvation temperature was set at 450 °C. Nitrogen gas was used as the nebulization gas at a flow rate of 800 L/h. The MassLynx XS™ 4.1 software (Waters Corporation, Manchester, UK) was used for the pre-processing and pre-treatment of the obtained raw data. The raw data files, together with the study description, have been deposited onto the online data repository, MetaboLights, an open-access general-purpose repository for metabolomics studies and associated meta-data, (www.ebi.ac.uk/metabolights/) with accession number MTBLS559.

### Multivariate data analysis

The MassLynx XS™ software parameters were set to analyse the 3–10 min retention time (Rt) range of the chromatograms, mass range 100–900 Da, mass tolerance 0.01 Da, mass window 0.05 Da and a Rt window of 0.20 min. The MarkerLynx™ data matrix (of Rt-*m/z* variable pairs, with the *m/z* peak intensities for each sample), was exported to the SIMCA (Soft Independent Modeling of Class Analogy) software, version 14 (Umetrics, Umeå, Sweden) for principal component analysis (PCA) using *Pareto* scaling and orthogonal projection to latent structures discriminant analysis (OPLS-DA) modeling also using *Pareto* scaling. PCA is an explorative non-biased model that reduces the multi-dimensionality of the complex raw data matrix obtained from the analysis whereas OPLS-DA is a biased model that allows for the extraction of the metabolites responsible between the separations of the differentially treated groups (e.g. control vs. treated) [22–24]. The selected mass ions from the OPLS-DA-derived S-plots and Variable Importance in Projection (VIP) plots were putatively annotated using the spectral fragmentation patterns (compared to literature) as well as the calculated putative empirical formulae and structures, which were further searched in databases such as the Dictionary of Natural Products (www.dnp.chemnet base.com), ChemSpider (www.chemspider.com) and Knapsack (http://kanaya.naist.jp/KNApSAcK/). Relative quantification was achieved using the SPSS software (http://www-01.ibm.com/software/za/analytics/spss/) to generate box-and-whiskers plots of relative peak intensity against time points.

### Total RNA extraction

Following elicitation, total RNA was extracted from harvested cells (100 mg) using the Trizol-reagent method (Invitrogen, Carlsbad, CA, USA). The extracted RNA samples were subjected to DNase treatment using DNase I (Thermo Scientific, Waltham, MA, USA). Concentrations were determined using a NanoDrop® ND-1000™Spectrophotometer (NanoDrop Inc., Wilmington, DE, USA). The RNA integrity of all samples was examined by electrophoresis on a 1.5% agarose gel in 1X Tris-Borate-EDTA (TBE) buffer containing 0.5 μg/mL ethidium bromide before use. The gels were visualized under UV light using a Bio-Rad Image Analyzer and Quantity One™ Version 4.6.1 Software (Bio-Rad Laboratories, Johannesburg, South Africa). The total RNA samples were divided into aliquots and stored at −80 °C for later use.

### Quantification of the expression of target genes

Real time PCR (qPCR) was used for the gene expression analysis [7, 67]. Prior to quantification of gene expression levels, the DNase-treated RNA isolated from cells harvested at the different time points were reverse-transcribed to cDNA using a RevertAid™ Premium First Strand cDNA synthesis kit (Fermentas, Thermo Scientific, Waltham, MA, USA). The selected genes included: *Phenylalanine ammonia lyase* (PAL), *Hydroxycinnamoyl-CoA:quinate hydroxycinnamoyl transferase* (HQT)*, Hydroxycinnamoyl-CoA:shikimate/quinate hydroxycinnamoyl transferase* (HCT), *Ethylene response element-binding protein* (EREBP)*, Small Sar1 GTPase* (SAR1-GTPase), *Non-expressor of PR genes 1* (NPR1)*, Thioredoxin* (THIO), *Heat shock protein 90* (HSP90)*, Required for Mla12 Resistance* (RAR1)*, Suppressor of the G2 allele of Skp1* (SGT1), *Pathogenesis-related protein 1a* (PR-1a) and *Defensin*. The choice of genes was based on our previous study where the response of tobacco cells towards isonitrosoacetophenone (INAP), a chemical inducer of defense responses [6], was investigated at transcriptome level [7]. The primer pairs [7] were designed using the ‘Primer Quest’ tool (Integrated DNA Technologies, Coralville, IA, USA) from sequences obtained in on-line data bases (GenBank NCBI, www.ncbi.nlm.nih.gov/genbank/). qPCR was performed to analyze the expression of each gene using a Rotor Gene-3000A instrument (Qiagen, Venlo, Netherlands) and the SensiFAST SYBR No-ROX Kit (Bioline, London, UK) according to the manufacturer’s instructions. Ten μL of SensiFAST SYBR, 1 μL forward primer (1 μM final concentration), 1 μL reverse primer (1 μM final concentration) and 6 μL of DNase-free water were added to 2 μL of cDNA for amplification in a total volume of 20 μL. The cycling conditions were as follows: initial denaturation for 10 min at 95 °C followed by amplification and quantification cycle repeated 40 times each consisting of 5 s denaturing at 95 °C, 10 s annealing at primer-specific temperatures, 20 s extension at 72 °C. The experimental design included three biological repeats with two technical repeats of each. Quantification of the relative changes in gene expression was performed using the relative standard curve method with *elongation factor 1-alpha* and *actin 8* as references genes [7]. Data sets were statistically compared between non-treated samples and treated samples at each time point using one-way analysis of variation (ANOVA) with the statistical analysis software GraphPad inStat 3 (GraphPad software, San Diego, CA, USA). The confidence level of all analyses was set at 95%, and values with *p* < 0.05 were considered significant.

## Additional files

Additional file 1: Figure S1. UHPLC-MS (ESI negative) base peak intensity (BPI) chromatograms of azelaic acid-treated *Nicotiana tabacum* cell extracts at 24 h (a), 12 h (b), 6 h (c) and 0 h (d) post-treatment. The most intense peaks at a specific retention time (Rt) are indicated with *m/z* values. (TIFF 84 kb)

Additional file 2: Figure S2. UHPLC-MS (ESI negative) base peak intensity (BPI) chromatograms of hexanoic acid-treated *Nicotiana tabacum* cell extracts at 24 h (a), 12 h (b), 6 h (c) and 0 h (d) post-treatment. The most intense peaks at a specific retention time (Rt) are indicated with *m/z* values. (TIFF 299 kb)

Additional file 3: Figure S3. Structures of annotated biomarkers: (1) caffeoylputrescine glycoside, (2) *cis*-5-caffeoylquinic acid, (3) feruloylglycoside, (4) azelaic acid glycoside, (5) feruloyl-3-methyltyramine glycoside, (6) feruloyl-3-methyltyramine conjugate and (7) feruloyl-3-methyltyramine. (TIFF 25 kb)

Additional file 4: Figure S4. UHPLC-MS spectra showing MS/MS fragmentation patterns for biomarkers 1–4. (TIFF 615 kb)

Additional file 5: Figure S5. UHPLC-MS spectra showing MS/MS fragmentation patterns for biomarkers 5–7. (TIFF 574 kb)

## Abbreviations

Aza: Azelaic acid
BPI: Base peal intensity
CGAs: Chlorogenic acids
CV Anova: Cross-validated analysis of variance
EREBP: Ethylene response element-binding protein
ESI: Electrospray ionization
ET: Ethylene
HCA: Hydroxycinnamic acid
HCT: Hydroxycinnamoyl-CoA:shikimate/quinate hydroxycinnamoyl transferase
HQT: Hydroxycinnamoyl-CoA:quinate hydroxycinnamoyl transferase
HSP90: Heat shock protein 90
Hxa: Hexanoic acid
ISR: Induced systemic resistance
JA: Jasmonic acid
M/PAMPs: Microbial/pathogen-associated molecular patterns
NB-LRR: Nucleotide-binding leucine-rich repeats
NPR1: Non-expressor of PR genes 1
OPLS-DA: Orthogonal projection to latent structures discriminant analysis
PAL: Phenylalanine ammonia lyase
PCA: Principal component analysis
PR-1a: Pathogenesis-related protein 1a
qRT-PCR: quantitative real-time PCR
RAR1: Required for Mla12 resistance
SA: Salicylic acid
SAR: Systemic acquired resistance
SAR1-GTPase: Small Sar1 GTPase
SGT1: Suppressor of the G2 allele of Skp1
SIMCA: Soft independent modelling of class analogy
THIO: Thioredoxin
UHPLC-Q-TOF/MS: Ultra high performance liquid chromatography quadrupole time of flight mass spectrometry
VIP: Variable importance in projection
XIC: Extracted ion chromatogram

## References

[1] Jones JDG, Dangl JL (2006). The plant immune system. Nature.

[2] Pieterse CMJ, Leon-Reyes A, van der Ent S, van Wees SCM (2009). Networking by small-molecule hormones in plant immunity. Nature.

[3] Sanabria NM, Huang J-C, Dubery IA (2010). Self/non-self-perception in plants in innate immunity and defense. Self/Non-Self Imm Recog Signal.

[4] Spoel SH, Dong X (2012). How do plants achieve immunity? Defence without specialized immune cells. Nat Rev Immunol.

[5] Choudhary DK, Prakash A, Johri BN (2007). Induced systemic resistance (ISR) in plants: mechanism of action. Indian J Med Microbiol.

[6] Madala NE, Steenkamp PA, Piater LA, Dubery IA (2014). Metabolomic insights into the bioconversion of isonitrosoacetophenone in *Arabidopsis thaliana* and its effects on defense-related pathways. Plant Physiol Biochem.

[7] Djami-Tchatchou AT, Maake MP, Piater LA, Dubery IA (2015). Isonitrosoacetophenone drives transcriptional reprogramming in *Nicotiana tabacum* cells in support of innate immunity and defense. PLoS One.

[8] Pastor V, Balmer A, Gamir J, Flors V, Mauch-Mani B (2014). Preparing to fight back: generation and storage of priming compounds. Front Plant Sci.

[9] Mhlongo MI, Piater LA, Steenkamp PA, Madala NE, Dubery IA (2014). Priming agents of plant defence stimulate the accumulation of mono- and di-acylated chlorogenic acids in cultured tobacco cells. Physiol Mol Plant Path..

[10] Martinez-Medina A, Flors V, Heil M, Mauch-Mani B, Pieterse CMJ (2016). Recognizing plant defense priming. Trends Plant Sci.

[11] Návarová H, Bernsdorff F, Döring AC, Zeier J (2012). Pipecolic acid. An endogenous mediator of defense amplification and priming, is a critical regulator of inducible plant immunity. Plant Cell.

[12] Llorens E, García-Agustín P, Lapeña L (2017). Advances in induced resistance by natural compounds: towards new options for woody crop protection. Sci Agric.

[13] Mauch-Mani B, Baccelli I, Estrella L, Flors V (2017). Defense priming: an adaptive part of induced resistance. Ann Rev Plant Biol.

[14] Aranega-Bou P, Leyva MO, Finiti I, García-Agustín P, González-Bosch C (2014). Priming of plant resistance by natural compounds. Hexanoic acid as a model. Front Plant Sci.

[15] Shah J, Zeier J (2013). Long-distance communication and signal amplification in systemic acquired resistance. Front Plant Sci.

[16] Yu K, Soares JM, Mandal MK, Wang CX, Chanda B, Gifford AN (2013). A feedback regulatory loop between G3P and lipid transfer proteins DIR1 and AZI1 mediates azelaic acid-induced systemic immunity. Cell Rep.

[17] Jung HW, Tschaplinski TJ, Wang L, Glazebrook J, Greenberg JT (2009). Priming in systemic plant immunity. Science.

[18] Vicedo B, Flors V, Leyva MO, Finiti I, Kravchuk Z, Real MD (2009). Hexanoic acid-induced resistance against *Botrytis cinerea* in tomato plants. Mol Plant-Microbe Interact.

[19] Kravchuk Z, Vicedo B, Flors V, Camañes G, González-Bosch C, García-Agustín P (2011). Priming for JA-dependent defenses using hexanoic acid is an effective mechanism to protect Arabidopsis against *B. cinerea*. J Plant Physiol.

[20] Clifford MN, Johnston KL, Knight S, Kuhnert N (2003). Hierarchical scheme for LC-MS^n^ identification of chlorogenic acids. J Agric Food Chem.

[21] Jaiswal R, Halabi EA, Karar MGE, Kuhnert N (2014). Identification and characterisation of the phenolics of *Ilex glabra* L. gray (Aquifoliaceae) leaves by liquid chromatography tandem mass spectrometry. Phytochemistry.

[22] Trygg J, Holmes E, Lundstedt T (2007). Chemometrics in metabonomics. J Proteome Res.

[23] Wiklund S, Johansson E, Sjoestroem L, Mellerowicz EJ, Edlund U, Shockcor JP (2008). Visualization of GC/ TOF-MS-based metabolomics data for identification of biochemically interesting compounds using OPLS class models. Anal Chem.

[24] Eriksson L, Trygg J, Wold S (2008). CV-ANOVA for significance testing of PLS and OPLS® models. J Chemomet.

[25] Galindo-Prieto B, Eriksson L, Trygg J (2014). Variable influence on projection (VIP) for orthogonal projections to latent structures (OPLS). J Chemomet..

[26] Saccenti E, Hoefsloot HCJ, Smilde AK, Westerhuis JA, Hendriks MMWB (2014). Reflections on univariate and multivariate analysis of metabolomics data. Metabolomics.

[27] Mhlongo MI, Piater LA, Steenkamp PA, Madala NE, Dubery IA (2016). Phenylpropanoid defences in *Nicotiana tabacum* cells: overlapping metabolomes indicate common aspects to priming responses induced by lipopolysaccharides, chitosan and flagellin-22. PLoS One.

[28] Mhlongo MI, Steenkamp PA, Piater LA, Madala NE, Dubery IA (2016). Profiling of altered metabolomic states in *Nicotiana tabacum* cells induced by priming agents. Front Plant Sci.

[29] Clifford MN (2000). Chlorogenic acids and other cinnamates - nature, occurrence, dietary burden, absorption and metabolism. J Sci Food Agr.

[30] Mhlongo MI, Piater LA, Steenkamp PA, Madala NE, Dubery IA (2015). Metabolomic fingerprinting of primed tobacco cells provide the first evidence for the biological origin of *cis*-chlorogenic acid. Biotechnol Lett.

[31] Conrath U (2009). Priming of induced plant defense responses. Adv Bot Res.

[32] Balmer A, Pastor V, Gamir J, Flors V, Mauch-Mani B (2015). The ‘prime-ome’: towards a holistic approach to priming. Trends Plant Sci.

[33] Bhalla R, Narasimhan K, Swarup S (2005). Metabolomics and its role in understanding cellular responses in plants. Plant Cell Rep.

[34] Dettmer K, Aronov PA, Hammock BD (2007). Mass spectrometry-based metabolomics. Mass Spectrom Rev.

[35] Tugizimana F, Piater L, Dubery I (2013). Plant metabolomics: a new frontier in phytochemical analysis. S Afr J Sci.

[36] Hall RD (2006). Plant metabolomics: from holistic hope, to hype, to hot topic. New Phytol.

[37] Allwood JW, De Vos RCH, Moing A, Deborde C, Erban A, Kopka J (2011). Plant metabolomics and its potential for systems biology research: background concepts, technology, and methodology. Methods Enzymol.

[38] Hayat Q, Hayat S, Irfan M, Ahmad A (2010). Effect of exogenous salicylic acid under changing environment: a review. Env Exp Bot.

[39] Tugizimana F, Steenkamp PA, Piater LA, Dubery IA (2014). Multi-platform metabolomic analyses of ergosterol-induced dynamic changes in *Nicotiana tabacum* cells. PLoS One.

[40] Finnegan T, Steenkamp PA, Piater LA, Dubery IA (2016). Activation of phytoanticipin and phytoalexin pathways in *Arabidopsis thaliana* in response to LPS elicitation: a metabolomic study. PLoS One.

[41] James JT, Tugizimana F, Steenkamp PA, Dubery IA (2013). Metabolomic analysis of methyl jasmonate-induced triterpenoid production in the medicinal herb *Centella asiatica* (L.) urban. Molecules.

[42] Allwood JW, Ellis DI, Goodacre R (2008). Metabolomic technologies and their application to the study of plants and plant–host interactions. Physiol Plant.

[43] Vogt T (2010). Phenylpropanoid biosynthesis. Mol Plant.

[44] Horbowicz M, Wiczkowski W, Koczkodaj D, Saniewski M (2011). Effects of methyl jasmonate on accumulation of flavonoids in seedlings of common buckwheat (*Fagopyrum esculentum Moench*). Act Biol Hung.

[45] López-Gresa MP, Torres C, Campos L, Lisón P, Rodrigo I, Bellés JM (2011). Identification of defence metabolites in tomato plants infected by the bacterial pathogen *Pseudomonas syringae*. Environ Exp Bot.

[46] Tohge T, Watanabe M, Hoefgen R, Fernie AR (2013). The evolution of phenylpropanoid metabolism in the green lineage. Crit Rev Biochem Mol Biol.

[47] Meurer-Grimes B, Berlin J, Strack D (1989). Hydroxycinnamoyl-CoA: putrescine hydroxycinnamoyltransferase in tobacco cell cultures with high and low levels of caffeoylputrescine. Plant Physiol.

[48] Wyss-benz M, Streit L, Ebert E (1990). Feruloylputrescine and caffeoylputrescine are not involved in growth and floral bud formation of stem explants from *Nicotiana tabacum* L. var Xanthi nc. Plant Physiol.

[49] Clifford MN, Kirkpatrick J, Kuhnert N, Roozendaal H, Salgado PR (2008). LC-MS^n^ analysis of the cis isomers of chlorogenic acids. Food Chem.

[50] Kong J-Q (2015). Phenylalanine ammonia-lyase, a key component used for phenylpropanoids production by metabolic engineering. RSC Adv.

[51] Niggeweg R, Michael AJ, Martin C (2004). Engineering plants with increased levels of the antioxidant chlorogenic acid. Nature. Biotechnol.

[52] Sonnante G, D’Amore R, Blanco E, Pierri CL, De Palma M, Luo J (2010). Novel Hydroxycinnamoyl-coenzyme a quinate transferase genes from artichoke are involved in the synthesis of chlorogenic acid. Plant Physiol.

[53] Hoffmann L, Besseau S, Geoffroy P, Ritzenthaler C , Meyer D, Lapierre C, et al. Silencing of hydroxycinnamoyl-coenzyme a shikimate/quinate hydroxycinnamoyltransferase affects phenylpropanoid biosynthesis. Plant Cell 2004; 16:1446–1465. 10.1105/tpc.020297 PMC490038 15161961

[54] Sanabria NM, van Heerden H, Dubery IA (2012). Molecular characterization and regulation of a *Nicotiana tabacum* S-domain receptor-like kinase gene induced during an early rapid response to lipopolysaccharides. Gene.

[55] New S-A, Piater LA, Dubery IA (2015). *silico* characterization and expression analysis of selected Arabidopsis receptor-like kinase genes responsive to different MAMP inducers. Biol Plant.

[56] Oosten VV, Van Loon LC, Mauch-Mani B, Turlings TCJ, Pieterse CMJ (2009). Priming as a mechanism behind induced resistance against pathogens, insects and abiotic stress. Induced resistance in plants against insects and diseases. IOBC/wprs Bull.

[57] Wang KLC, Li H, Ecker JR (2002). Ethylene biosynthesis and signalling networks. Plant Cell.

[58] Pei H, Sun Q, Hao Q, Lv B, Wu J, Fu D (2015). The HSP90-RAR1-SGT1 based protein interactome in barley and stripe rust. Physiol Mol Plant Path..

[59] Seo YS, Lee SK, Song MY, Suh JP, Hahn TR (2012). The HSP90 complex of plants. Biochim Biophys Acta.

[60] Mou Z, Fan W, Dong X (2003). Inducers of plant systemic acquired resistance regulate NPR1 function through redox changes. Cell.

[61] Dong XN (2004). NPR1, all things considered. Curr Opin Plant Biol.

[62] Thomma BP, Eggermont K, Penninckx IA, Mauch-Mani B, Vogelsang R, Cammue B, Broekaert WF (1998). Separate jasmonate-dependent and salicylate-dependent defense response pathways in Arabidopsis are essential for resistance to distinct microbial pathogens. Proc Natl Acad Sci U S A.

[63] Berndt C, Lillig CH, Holmgred A (2008). Thioredoxins and glutaredoxins as facilitators of protein folding. Biochim Biophys Acta.

[64] Van Loon LC, Van Strien EA (1999). The families of pathogenesis-related proteins, their activities, and comparative analysis of PR-1 type proteins. Physiol Mol Plant Path.

[65] Takahashi H, Kanayama Y, Zheng MS, Kusano T, Hase S, Ikegami M (2004). Antagonistic interactions between the SA and JA signaling pathways in Arabidopsis modulated expression of defense genes and gene for gene resistance to cucumber mosaic virus. Plant Cell Physiol.

[66] Tugizimana F, Steenkamp PA, Piater LA, Dubery IA (2012). Ergosterol-induced sesquiterpenoid synthesis in tobacco cells. Molecules.

[67] Derveaux S, Vandesompele J, Hellemans J (2010). How to do successful gene expression analysis using real-time PCR. Methods.

[68] Haug K, Salek RM, Conesa P, Hastings J, de Matos P (2013). MetaboLights–an open-access general-purpose repository for metabolomics studies and associated meta-data. Nucleic Acids Res.

