# Supplementary figures and images for: Similar, but different: structurally related azelaic acid and hexanoic acid trigger differential metabolomic and transcriptomic responses in tobacco cells
Source: BMC Plant Biol. 2017 Nov 29;17:227. doi: 10.1186/s12870-017-1157-5 (PMC5706331; doi:10.1186/s12870-017-1157-5)

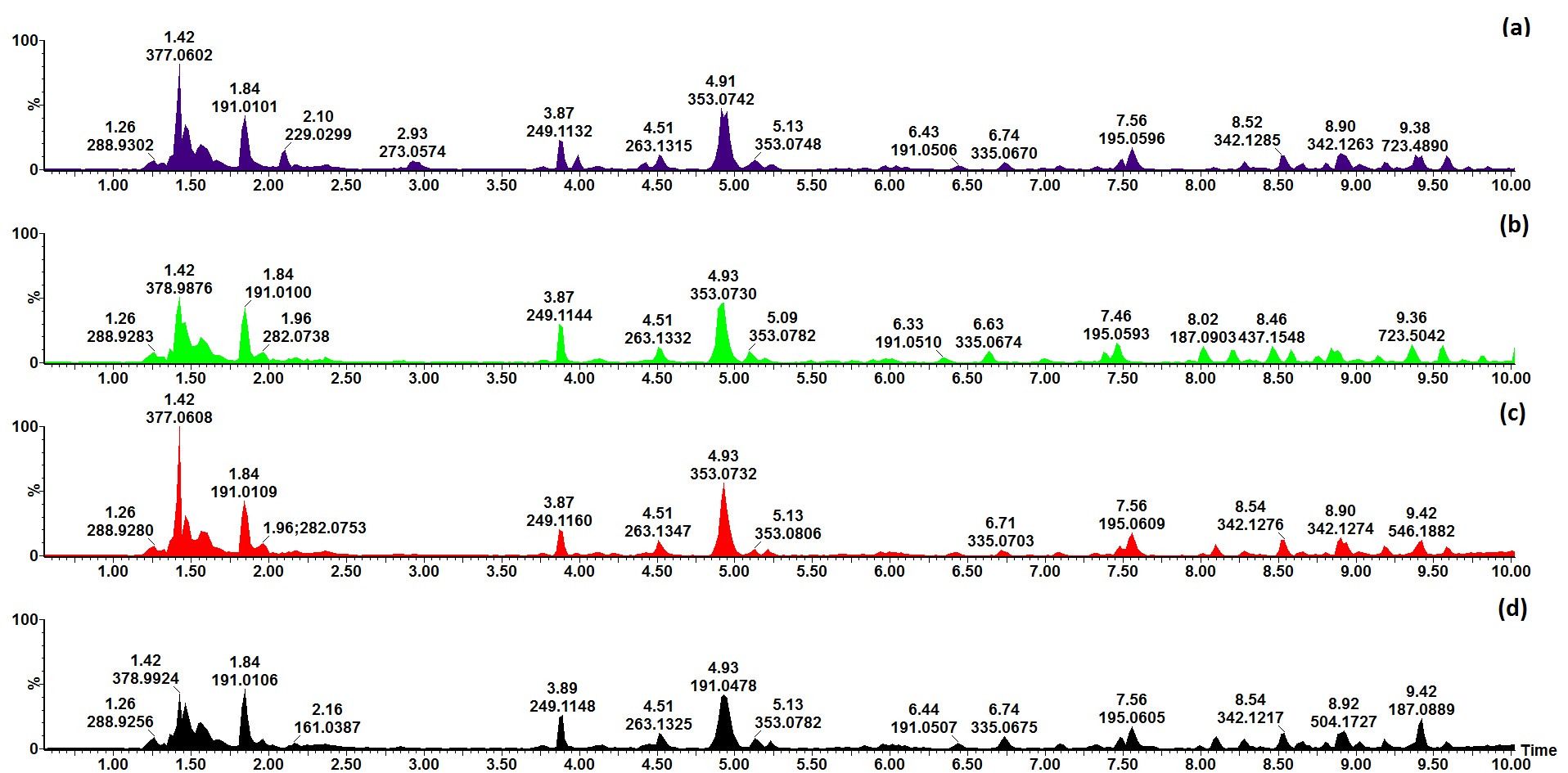

Supplement: Supplementary file 1 — UHPLC-MS (ESI negative) base peak intensity (BPI) chromatograms of azelaic acid-treated Nicotiana tabacum cell extracts at 24 h (a), 12 h (b), 6 h (c) and 0 h (d) post-treatment. The most intense peaks at a specific retention time (Rt) are indicated with m/z values. (TIFF 84 kb) [file 12870_2017_1157_MOESM1_ESM.tif]

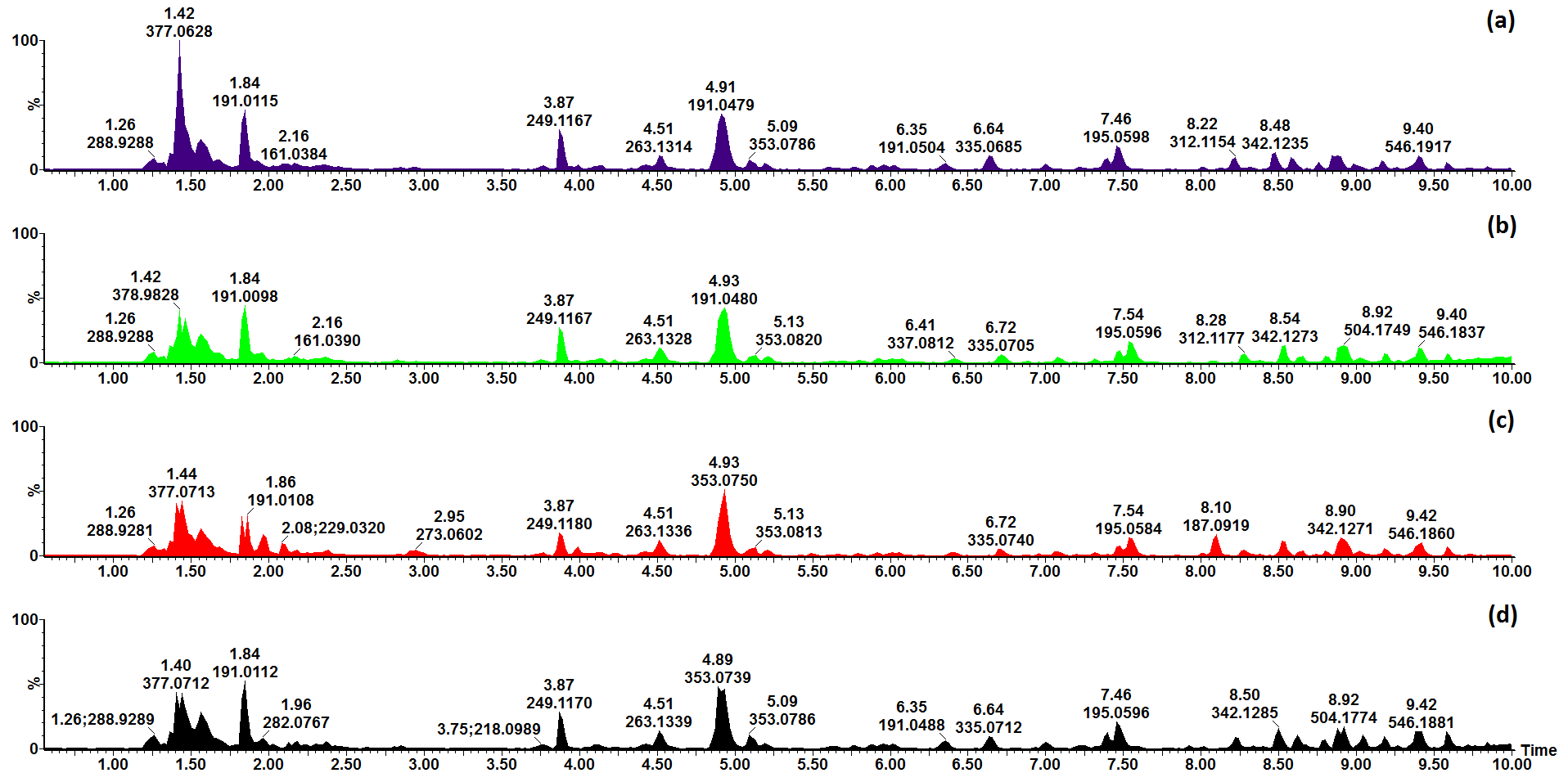

Supplement: Supplementary file 2 — UHPLC-MS (ESI negative) base peak intensity (BPI) chromatograms of hexanoic acid-treated Nicotiana tabacum cell extracts at 24 h (a), 12 h (b), 6 h (c) and 0 h (d) post-treatment. The most intense peaks at a specific retention time (Rt) are indicated with m/z values. (TIFF 299 kb) [file 12870_2017_1157_MOESM2_ESM.tif]

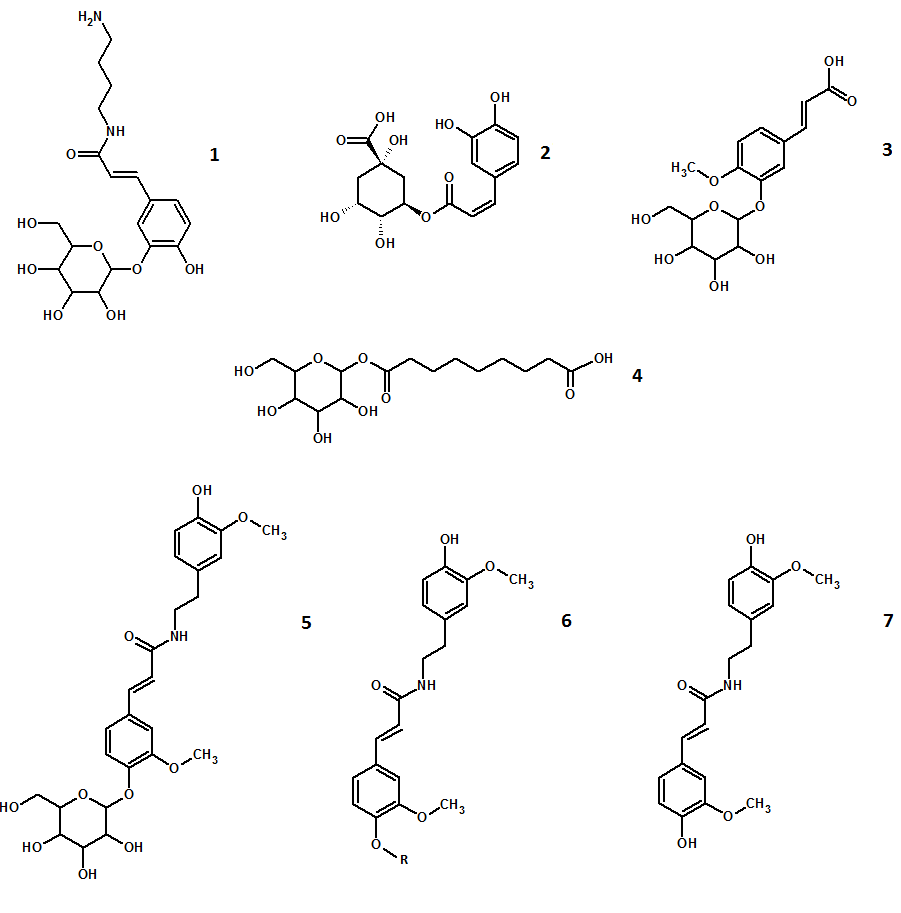

Supplement: Supplementary file 3 — Structures of annotated biomarkers: (1) caffeoylputrescine glycoside, (2) cis-5-caffeoylquinic acid, (3) feruloylglycoside, (4) azelaic acid glycoside, (5) feruloyl-3-methyltyramine glycoside, (6) feruloyl-3-methyltyramine conjugate and (7) feruloyl-3-methyltyramine. (TIFF 25 kb) [file 12870_2017_1157_MOESM3_ESM.tif]

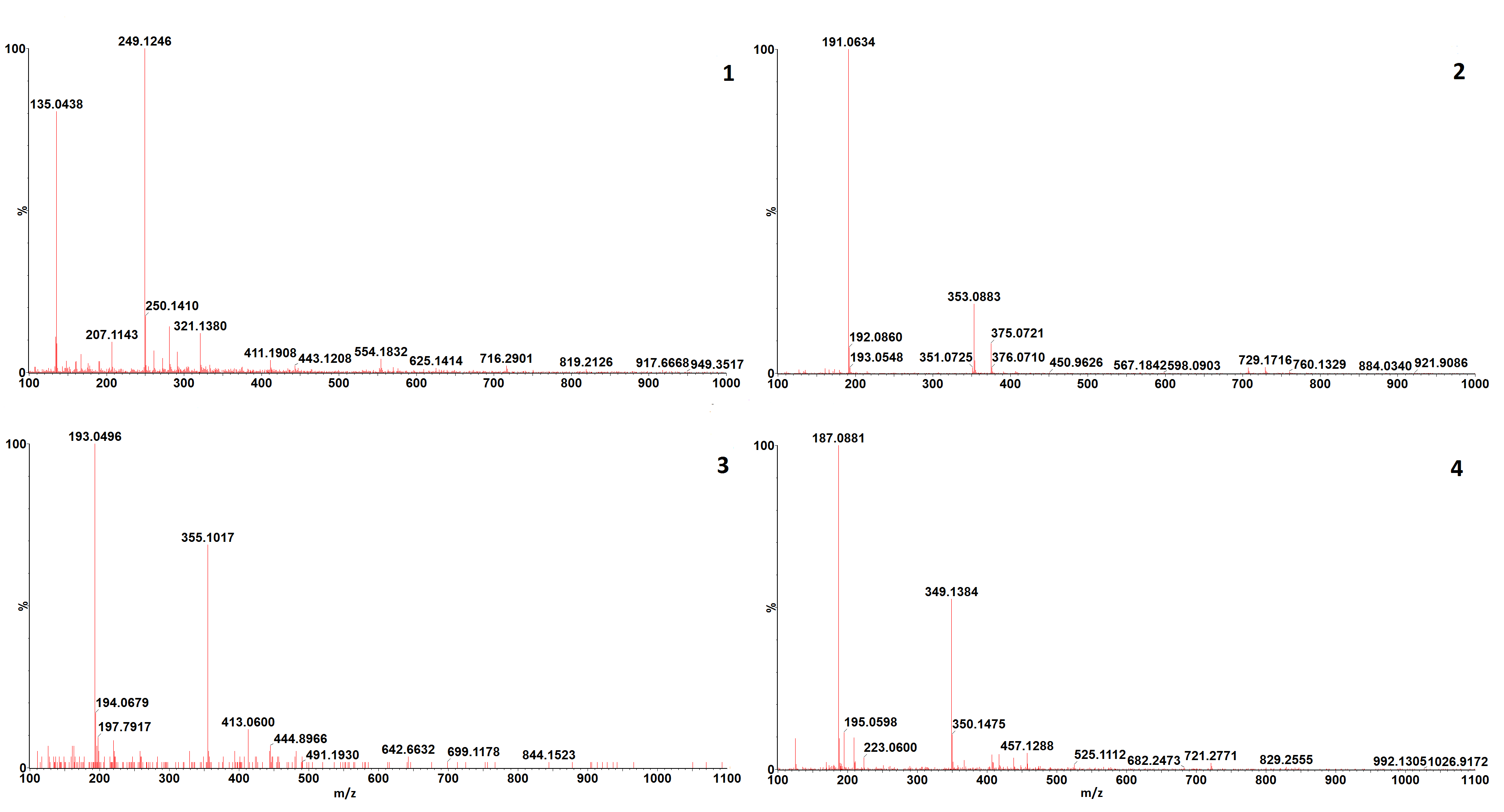

Supplement: Supplementary file 4 — UHPLC-MS spectra showing MS/MS fragmentation patterns for biomarkers 1–4. (TIFF 615 kb) [file 12870_2017_1157_MOESM4_ESM.tif]

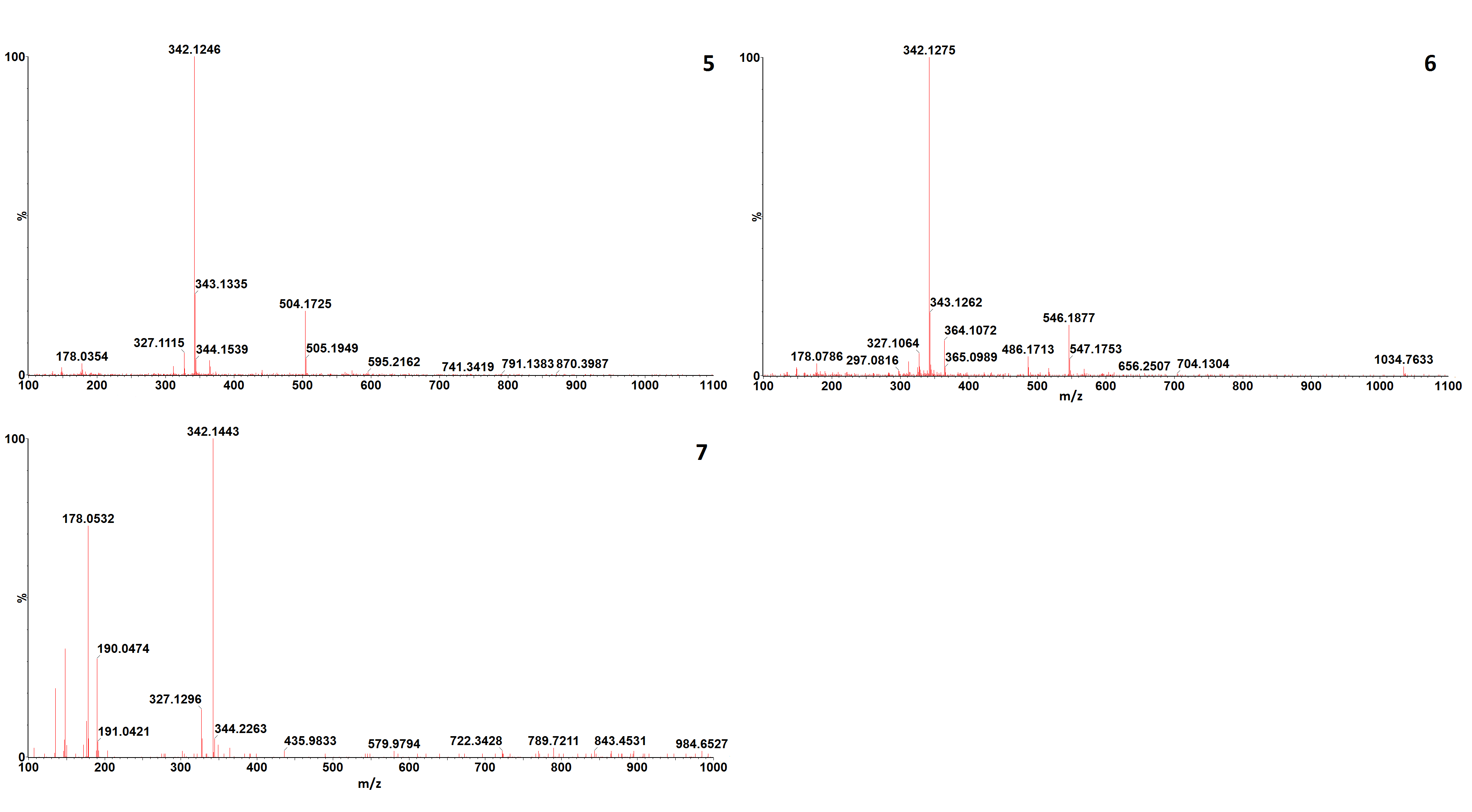

Supplement: Supplementary file 5 — UHPLC-MS spectra showing MS/MS fragmentation patterns for biomarkers 5–7. (TIFF 574 kb) [file 12870_2017_1157_MOESM5_ESM.tif]
